# An Improved Boundary-Aware U-Net for Ore Image Semantic Segmentation

**DOI:** 10.3390/s21082615

**Published:** 2021-04-08

**Authors:** Wei Wang, Qing Li, Chengyong Xiao, Dezheng Zhang, Lei Miao, Li Wang

**Affiliations:** 1 School of Automation and Electrical Engineering, University of Science and Technology Beijing, Beijing 100083, China; b20200301@xs.ustb.edu.cn (W.W.); liqing@ies.ustb.edu.cn (Q.L.); xcyustb@163.com (C.X.); lei259@ustb.edu.cn (L.M.); 2 Key Laboratory of Knowledge Automation for Industrial Processes, University of Science and Technology Beijing, Ministry of Education, Beijing 100083, China; 3 School of Computer and Communication Engineering, University of Science and Technology Beijing, Beijing 100083, China; zdzchina@126.com; 4 Beijing Key Laboratory of Knowledge Engineering for Materials Science, University of Science and Technology Beijing, Beijing 100083, China

**Keywords:** ore image segmentation, U-Net, improved encoder, multi-task learning, boundary mask fusion block

## Abstract

Particle size is the most important index to reflect the crushing quality of ores, and the accuracy of particle size statistics directly affects the subsequent operation of mines. Accurate ore image segmentation is an important prerequisite to ensure the reliability of particle size statistics. However, given the diversity of the size and shape of ores, the influence of dust and light, the complex texture and shadows on the ore surface, and especially the adhesion between ores, it is difficult to segment ore images accurately, and under-segmentation can be a serious problem. The construction of a large, labeled dataset for complex and unclear conveyor belt ore images is also difficult. In response to these challenges, we propose a novel, multi-task learning network based on U-Net for ore image segmentation. To solve the problem of limited available training datasets and to improve the feature extraction ability of the model, an improved encoder based on Resnet18 is proposed. Different from the original U-Net, our model decoder includes a boundary subnetwork for boundary detection and a mask subnetwork for mask segmentation, and information of the two subnetworks is fused in a boundary mask fusion block (BMFB). The experimental results showed that the pixel accuracy, Intersection over Union (IOU) for the ore mask (IOU_M), IOU for the ore boundary (IOU_B), and error of the average statistical ore particle size (ASE) rate of our proposed model on the testing dataset were 92.07%, 86.95%, 52.32%, and 20.38%, respectively. Compared to the benchmark U-Net, the improvements were 0.65%, 1.01%, 5.78%, and 12.11% (down), respectively.

## 1. Introduction

The particle size of ores is an important index to judge the completion degree of ore crushing. Statistical information regarding ore particle size can help determine whether there are large or special-shaped ores that will cause accidents at the mine site, such as blockage caused by large ores and scratches on the belt surface from special-shaped ores, to provide sufficient information for the control of ore crushing and transportation. Accurate ore image segmentation is the first step to obtaining reliable particle size information.

**Figure 1:**
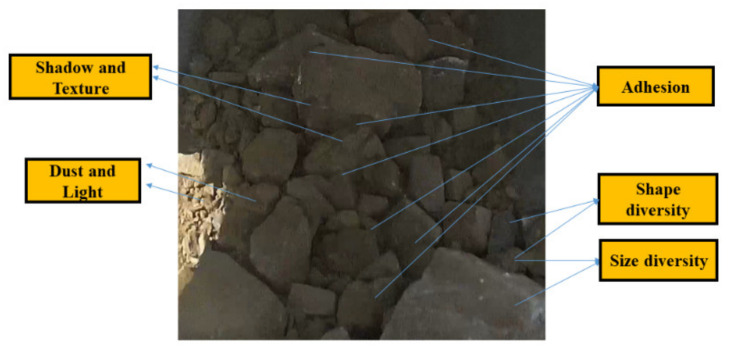
An example of ore images.

However, there are many obstacles to accurately segment ore images (e.g., the size and shape diversity of ores, the influence of dust and light, complex texture and shadows on the ore surface, and the adhesion between ores) as shown in Figure 1. Specifically, the adhesion between ores will cause the ore boundary to blur or even disappear, leading to under-segmentation.

The traditional ore particle size detection methods, including artificial screening and physical settlement, have strong adaptability and are widely used. However, they consume enormous human resources, and the detection results are easily affected by workers’ subjective factors. In recent years, automatic detection methods of ore particle size based on traditional image processing have been proposed, and there have been significant breakthroughs, including watershed and its improved methods [1,2,3,4], threshold segmentation method [5,6], and segmentation method based on specific theory [7] are the main measures. However, these methods rely on tedious parameter tuning processes to obtain satisfactory results and are difficult to generalize to different scenarios.

Since AlexNet [8] won the championship of the ImageNet Large-Scale Visual Recognition Challenge [9] in 2012, deep learning based on convolutional neural networks (CNNs) has made great breakthroughs in image classification, and even achieved a higher accuracy rate than human-level performance [10]. Benefitting from the development of image classification based on CNNs, object detection and semantic segmentation in the downstream field have also made significant progress. For example, the two-stage Faster R-CNN [11] has become the benchmark of object detection.

As for semantic segmentation, the best segmentation results of different scenes are from U-Net [12], PSP-Net [13], DeepLabv3+ [14], and their variants. In ore image segmentation, CNN-based techniques have also shown great advantages over traditional image processing methods [15]. Liu et al. [16] adopted U-Net to preliminarily segment ore images and then use Res-Unet to optimize the segmentation masks. The results showed that their two-step ore contour detection and segmentation optimization method obtained more accurate results compared with the one-step segmentation model.

Their work is quite similar to [17], and the drawback of their model is that more parameters are required, as the whole framework includes two separate models, and the final segmentation cannot be achieved in an end-to-end way. Li et al. [18] introduced their model based on U-Net, which was lighter through reducing the channel number and alleviated common over-segmentation and under-segmentation problems of ore image segmentation by optimizing the loss function and using the watershed algorithm. Their idea was referenced by [19].

Based on the understanding that belt ores have different shapes and sizes, Xiao et al. [20] used deformable convolution [21] to replace ordinary convolution, which greatly improved the ore image segmentation accuracy. In [22], an improved encoder-decoder network based on U-Net with a redesigned decoder and a flexible contour awareness loss was proposed for multi-class ore image segmentation.

The experiment showed that their proposed scheme achieved the best performance compared with counterparts (i.e., the Ternausnet in [23] and Attention U-Net in [24]). Ma et al. [25] proposed a new belt ore image segmentation method based on CNN and image processing technology. The results showed that a proper image preprocess was crucial to obtain a satisfactory prediction.

Here, we adopted a multi-task learning framework based on U-Net. Our model is a variant of [26,27,28]. Unlike the models based on fully convolutional networks (FCN) [29] in [26,27], we adopted U-Net as our benchmark due to its power in restoring boundary information by the encoder-decoder symmetrical structure and skip-connection, which met our need of accurate ore boundary segmentation.

In contrast to [28] in which feature maps from the boundary decoding path at each layer were concatenated with those from the main decoding path to improve the quality of cell segmentation, the information of our two decoding paths, the boundary subnetwork and the mask subnetwork, was fused in a boundary mask fusion block. To alleviate the effect of a limited training dataset and improve the feature extraction ability of our model, the original encoder of U-Net was replaced by an improved encoder based on Resnet18 [30] with pre-trained parameters from ImageNet.

Our main contributions are: (1) we are the first to apply a multi-task U-Net framework for ore image segmentation. (2) We proposed a novel boundary-aware U-Net with a boundary subnetwork for boundary detection and a mask subnetwork for mask segmentation, and the information of two subnetworks was fused in a boundary mask fusion block. (3) An improved encoder based on Resnet18 with pre-trained parameters from ImageNet was utilized to alleviate the data availability limitation. (4) We demonstrate that the proposed network improved the ore image segmentation accuracy.

The rest of this article is organized as follows: Section 2 introduces the acquisition and preprocessing methods of the ore image dataset. Section 3 describes the network structure of the boundary-aware U-Net. Section 4 presents the quantitative and qualitative experimental results. Finally, our conclusions are drawn in Section 5.

## 2. Acquisition and Preprocessing Methods of Dataset

Deep learning is known to be data-driven. The more data, the better one’s prediction results will be. Our ore images were derived from conveyor belt video data of Ansteel Mining. After selecting representative ore images, the professional software Labelme was used to annotate the raw images to obtain corresponding labels. The ore images had only two categories: ore and background. We ignored many tiny ores instead of labeling ores in images one by one.

**Figure 2:**
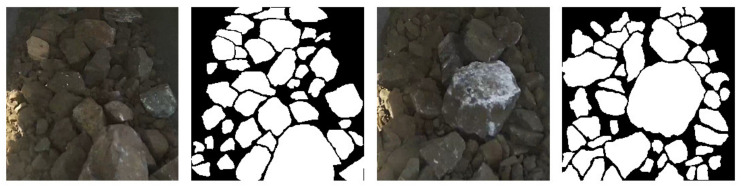
Examples of ore images and their annotations.

After obtaining binary images of the ore image labels, OpenCV was used to delete ore labels with pixels less than 2500, as segmenting tiny ores is not important, and labeling them is a harder and more time-consuming work than labeling normal ones. As for the CNN, the scale is one of the features that it can learn. Figure 2 shows two selected ore images and their annotation results.

Data preprocessing is the first and possibly the most critical step for the whole segmentation process, since proper preprocessing is of great benefit to the prediction results. Three ore image preprocessing methods were adopted in this paper. Firstly, to reduce the time of model training and prediction, we gray-scaled the ore images to reduce the data amount of the input images by two thirds. Secondly, considering there was excess noise on the ore surface, it was necessary to preprocess the images using fuzzy techniques [31].

**Figure 3:**
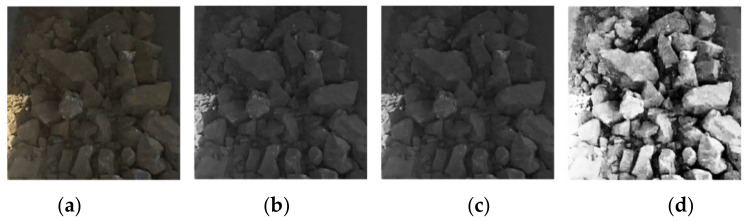
An example of raw image and its preprocessed results: (**a**) the raw image; (**b**) the gray scale image; (**c**) the bilateral filtering result; and (**d**) the contrast-limited adaptive histogram equalization (CLAHE) result.

We choose a bilateral filter to process the grayscale ore images for noise reduction and ore boundary information preservation at the same time. Lastly, contrast-limited adaptive histogram equalization (CLAHE) was used to enhance the contrast between the ore and background, as well as to make the boundary of ores more distinct. The raw image and its preprocessed results are shown in Figure 3.

## 3. Boundary-Aware U-Net

**Figure 4:**
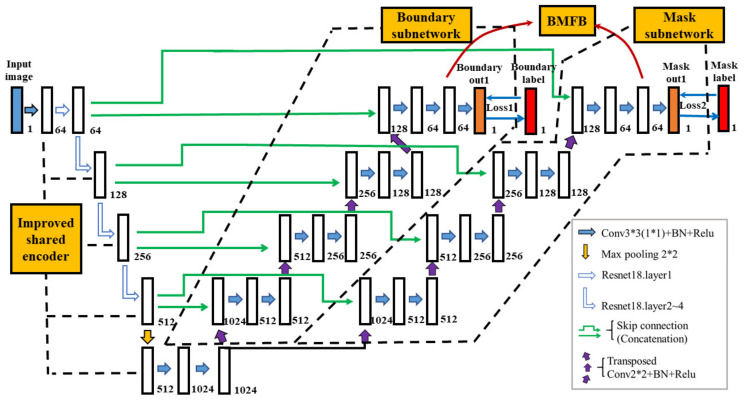
Network structure of the boundary-aware U-Net.

The overall structure of our network is shown in Figure 4, which consists of four parts: the encoder, boundary subnetwork decoder, mask subnetwork decoder, and boundary mask fusion block (BMFB). The encoder’s function is to extract abstract high-level semantic information of the input images for subsequent pixel binary classification. Ores on the conveyor belts are in a harsh environment, with the dust on the ore surface, the adhesion between ores, and the large number of ores in an image all forming an obstacle to ore manual annotation; therefore, it is difficult to obtain a large training dataset. Training with a small dataset, however, tends to result in overfitting.

Experiments [22,23] showed that models based on transfer learning can speed up the training process, suppress overfitting, and improve the accuracy of image segmentation. After experiment comparisons, the improved encoder based on Resnet18 was adopted in this paper. Different from the original Resnet18 in [30], we abandoned the first 7*7 convolution with stride 2 and the first max-pooling, so as not to down-sample four times by conducting only one convolution operation.

On the other hand, after the fourth layer of Resnet18, we conducted a max-pooling operation to down-sample the image resolution and added two convolution operations for further processing the semantic information obtained based on transfer learning and keeping the same 16 times down-sample rate as the original U-Net. As our input images were grayscale, a 3*3 convolution was performed before layer1 of Resnet18 to change the channel of feature maps from 1 to 64, while keeping the same image resolution. The detailed differences of the three encoders from our improved encoder, the original ResNet18 and original U-Net are shown in Table 1.

The bracket represents the Residual Block in [30]. Layer1_0 represents the first operation before layer1. The input size is 256 * 256, and the output size of each layer is shown in the second column. From left to right, the size results are from the three encoders, respectively. In addition, the three dashes mean no operation or no result. For expressions in the form of “a × a, b”, a represents the number of kernel size and b is the number of convolution kernel filters.

In contrast with the decoder of the original U-Net, our decoding path was divided into the boundary subnetwork, mask subnetwork, and boundary mask fusion block (BMFB). The boundary subnetwork was used to preliminarily detect the boundaries in ore images. The mask subnetwork was used to preliminarily predict the mask, and the last 64 feature maps of both subnetworks were sent to BMFB for feature fusion and the final mask prediction. Each subnetwork before BMFB was identical to the decoder of the original U-Net.

**Figure 5:**
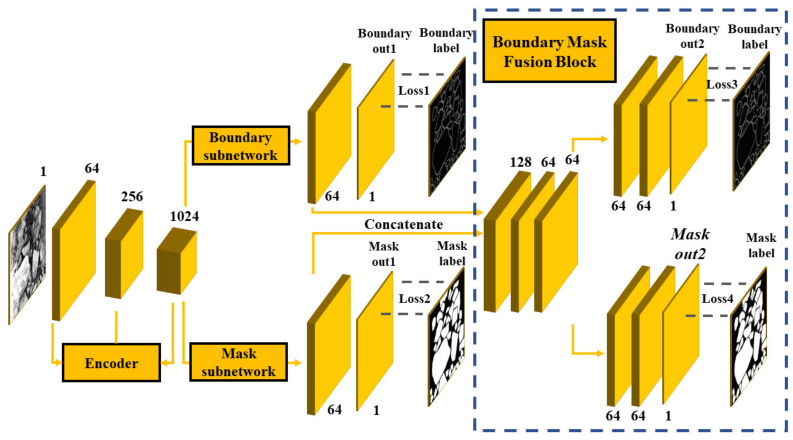
Simplified network structure and the detailed boundary mask fusion block (BMFB).

The boundary labels were obtained from mask labels by using the Canny edge detection algorithm in OpenCV. The simplified overall network architecture and the detailed BMFB are shown in Figure 5. Our decoder is different from [26], as their decoder was simply divided into two subnetworks without combining their information. Our decoder is also different from [27], whose idea was to use boundary information from the boundary subnetwork to help the BMFB obtain a more accurate mask segmentation.

Although they integrate feature maps of two subnetworks, only the mask was predicted in their BMFB, meaning that information from the mask subnetwork was not helpful in boundary prediction. Inspired by [32,33], we considered that boundary information is as important as mask information. Thus, in our BMFB, we had two subnetworks for predicting the boundary and mask, respectively. Only the final mask out2 was used for testing.

Our loss function comprises four parts, including boundary subnetwork Loss1, mask subnetwork Loss2, boundary Loss3, and mask Loss4 of BMFB. Loss1 is the same as Loss3, whose labels are the ore boundaries. Loss2 is identical to Loss4, whose labels are the ore masks. The reason for using Loss1 and Loss2 is to let the two subnetworks know what their goals are before BMFB. Since the label of each loss function has only two categories, ore (ore boundary) or background, a binary cross entropy was adopted for all of them. The loss function is expressed as follows:

(1) $$BCE=-\frac{1}{n}\overset{n}{\underset{i=1}{\sum }}\left(y_{i}\times \log p_{i}+\left(1-y_{i}\right)\times \log\left(1-p_{i}\right)\right)$$

where y*_i_* is the true label of each pixel, with 1 for foreground or 0 for background, while p*_i_* is the predicted probability of the pixel belonging to the foreground.

The pixel accuracy (Acc), F1 score, Intersection over Union for the ore mask (IOU_M), precision, recall, under-segmentation, over-segmentation, and Intersection over Union for the ore boundary (IOU_B) were chosen as our basic performance metrics on the testing dataset. Their formulas are as follows:

(2) $$Acc=\frac{TP+TN}{TP+TN+FP+FN}$$

(3) $$F1=\frac{2\times precison\times recall}{precision+recall}$$

(4) $$IOU\_M=\frac{TP}{TP+FP+FN}$$

(5) $$precison=\frac{TP}{TP+FP}$$

(6) $$recall=\frac{TP}{TP+FN}$$

(7) $$under\_seg=\frac{FP}{TN+FP}$$

(8) $$over\_seg=\frac{FN}{TP+FN}$$

(9) $$IOU\_B=\frac{TP}{TP+FP+FN}$$

where TP represents the number of pixels with both predicted value and label value 1, TN represents the number of pixels with both predicted value and label value 0, FP represents the number of pixels with predicted value of 1 and label value of 0, and FN represents the number of pixels with predicted value of 0 and label value of 1. The difference between IOU_M and IOU_B is that the foreground of the former one is the ore mask, while the foreground of the latter one is the ore boundary. The predicted ore boundary used for calculating IOU_B is not the boundary out2 in Figure 5 but the result obtained from the mask out2 using the Canny edge detection algorithm in OpenCV.

Inferencing [18], we adopt the error of the average statistical ore particle size (ASE) as the final performance metric. After obtaining the segmented ore image, we traverse the closed region in the image to obtain the area. Using the obtained region area 𝑆—the number of pixels belonging to a closed region in the segmented image—we can calculate the particle size 𝐷 by Equation (10):

(10) $$D=2\times \sqrt{\frac{S}{\pi }}$$

After finding the particle size, it is necessary to define the statistical interval. We use one pixel to represent 1 cm. Since the particle size D of a piece of ore is typically less than 160 cm, setting a statistical interval every 20 cm between 0 and 160 cm can meet the statistical tasks’ needs. Finally, the total amount of ores must be counted as a statistical interval as well.

Since the ultimate goal of ore particle size statistics is to count the cumulative size distribution, we calculated the average of all the test results, and the results of the annotation label images were used as a reference. In each interval, we count the absolute value of the difference between the statistical ore quantity of the predicted results and the actual labels and divided it by the latter. The ASE was obtained by calculating the mean of each interval. The formula of ASE is as follows:

(11) $$ASE=\frac{1}{M\times m}\overset{m}{\underset{i=1}{\sum }}\overset{M}{\underset{j=1}{\sum }}\frac{\left|N_{pre}^{j}-N_{label}^{j}\right|}{N_{label}^{j}}$$

where m is the number of the testing dataset and M is the total number of statistics intervals, 10 and 9 in this paper, respectively. N*_pre_* and N*_label_* are the numbers of ores that belong to interval j in the predicted images and the true labels, respectively.

## 4. Experiment Results

### 4.1. Dataset and Preprocessing

There were 86 ore images in our experiment, 76 of which were randomly selected as the training dataset, and the rest were chosen as the testing dataset. Offline, we flipped the training dataset images horizontally to amplify the numbers to 152. The original image resolution was 980 * 980, and we resized both the training dataset and testing dataset to 256 * 256.

Online, we normalized the training dataset making the mean value of the input images 0 and the standard deviation 1. In addition, in every training epoch, we used RandomResizedCrop in PyTorch to augment the training dataset. The interval of scaling and aspect ratio were both 0.8–1.2, and the resized size was 256 to increase the multi-scale information of the input ore images. For the testing dataset, only the normalization operation was conducted.

### 4.2. Experiment Details

The experiment was carried out on a Windows10 operation system. The programming language was python3.6, and the deep learning framework was PyTorch. The graphics cards were two Nvidia GeForce GTX 1080 with 8G memory, and the CUDA version was 10.2. Except for the pre-trained parameters from ImageNet, other parameters were initialized by the Kaiming initialization method [10]. The optimizer selected was Adam [34]. The learning rate was set to 0.0001. The number of training epochs was 300. The model with the highest pixel accuracy on the training dataset was saved for prediction, and a random seed was fixed for fair experiment comparisons and reproduction.

### 4.3. Result Analysis

At first, comparison experiments were conducted to verify the effectiveness of our designed multi-task double subnetworks decoder with BMFB (dd for short) and the improved encoder based on ResNet18 (res18 for short). The original U-Net was used as the benchmark. “+dd” and “+res18” indicate that the model’s decoder is our redesigned dd and the encoder is our improved res18, respectively.

The “+res18” also indicates that we only adopted the network structure whose initial parameters were all from the Kaiming initialization method. In contrast “+param” indicates that the pre-trained parameters from ImageNet were used for the layers 1 to 4 of the encoder. The quantitative performance on the testing dataset is shown in Table 2.

**Figure 6:**
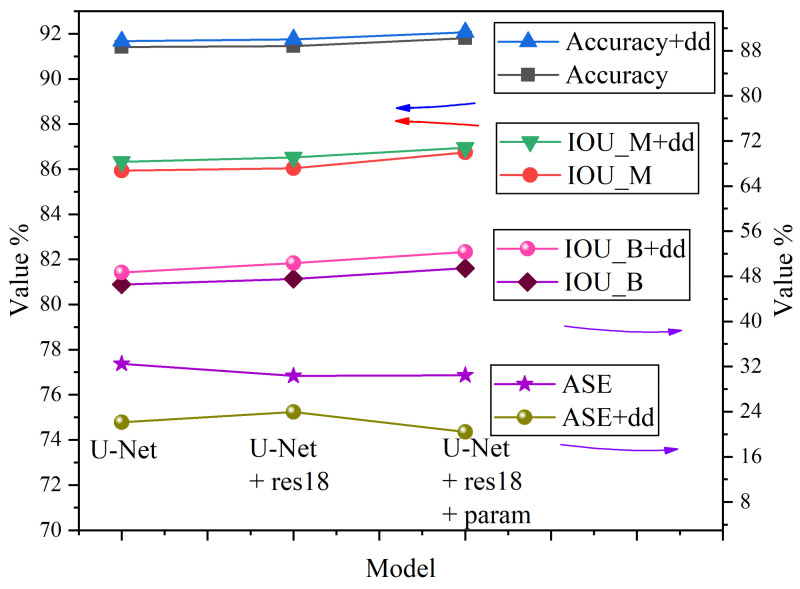
Comparison among different U-Net based models.

The pixel accuracy, IOU_M, IOU_B, and ASE of different models, which are the most representative and important performance metrics, were selected to draw Figure 6. From the abscissa of Figure 6, as the model encoder was replaced by res18, the pixel accuracy, IOU_M, and IOU_B were all improved. With the adoption of pre-trained parameters from ImageNet, the three performance metrics were further improved.

The ASE of the three models without dd decreased firstly and then maintained a steady-state, while the ASE of the three models with dd increased slightly and then decreased to the bottom. On the other hand, compared with models with the decoder of the original U-Net, the pixel accuracy, IOU_M, IOU_B of models with dd were improved, and the ASE rate was decreased by a wide margin. For example, the ASE of U-Net+dd was 22.13%, which was 10.36% lower than that of U-Net, proving the advantage of the proposed dd designation with BMFB in segmenting adhesive ores.

**Figure 7:**
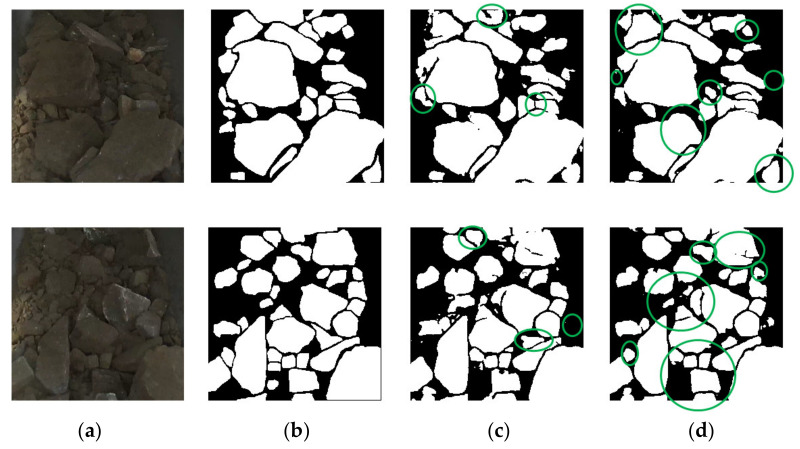
Predictions of U-Net and U-Net+dd: (**a**) the raw image; (**b**) the ground truth; (**c**) the U-Net result; and (**d**) the U-Net+dd result.

The prediction results of U-Net and U-Net+dd are shown in Figure 7, where green boxes represent better predictions. U-Net+dd was more conducive to segmentation of adhesive ores. The U-Net+res18+param+dd model had another 3.46% higher IOU_B and a 1.75% lower ASE rate compared with that of U-Net+dd, while exhibiting the highest pixel accuracy rate and IOU_M; therefore, it was chosen as our final model. The pixel accuracy rate of our model was 92.07%, the IOU_M was 86.95%, the IOU_B was 52.36%, and the ASE rate was 20.38% on the testing dataset.

Compared to the benchmark U-Net, the improvements were 0.65%, 1.01%, 5.78%, and 12.11% (down), respectively. In fact, our model performed better in segmenting adhesive ores since it can recognize ore boundary pixels more accurately. However, ore boundary pixels occupy a tiny proportion in the whole image compared with ore mask pixels, and thus only a 1.01% improvement in IOU_M was observed. On the other hand, the IOU_M of the baseline model U-Net was quite high, which made improving it difficult.

Even our improved encoder res18 based on transfer learning only achieved 0.8% improvement, and our designed dd decoder had an improvement of 0.49% when adopted independently. The improvements from our solo res18+param and solo dd to IOU_B were 2.9% and 2.32%, respectively, and the improvement reached 5.78% when combining them. Once the ore boundary can be detected more accurately, the problem of under-segmentation can be alleviated, which was reflected in the reduction of the ASE from 32.49% of U-Net to 20.38% of U-Net+res18+param+dd.

In the design of the encoder, we conducted comparison experiments of different encoders (i.e., original U-Net, VGG16 [35], Resnet18 [30], our res18, and the improved encoder based on Resnet34 (res34)). The difference between res18 and res34 was the same as that in [30], the repeated times of the Residual block in res18 at each layer were [2,2,2,2], compared to [3,3,4,6] in res34. The reason why no improved encoder based on Resnet50 was designed is that its channel number of the last feature map at each layer is three-times larger than that of Resnet34 or Resnet18. 

If we keep each Resnet50′s decoder symmetric with the encoder, the parameters of the whole model will be extremely large. The models with encoders from the original VGG16 and Resnet18 are named U-Net+VGG16 [35] and U-Net+Res18 [30], respectively. The models using our res18 encoder and res34 encoder are named U-Net+res18 (ours) and U-Net+res34 (ours), respectively. All four models mentioned above were initialized with pre-trained parameters from ImageNet and dd was adopted.

Since our input is grayscale images, a 3*3 convolution was performed at the first step to convert the channel from 1 to the number that can be directly used in the pre-trained model: 3 for U-Net+vgg16 [35] and U-Net+Res18 [30], and 64 for U-Net+res18 (ours) and U-Net+res34 (ours). The quantitative experiment results on the testing dataset are shown in Table 3.

As can be seen from Table 3, compared with the original U-Net, the U-Net+VGG16 [35] and U+Net+Res18 [30] with our dd, which were already proved to be effective, did not improve in most performance metrics on the testing dataset, especially for the pixel accuracy and IOU_M. The U-Net+VGG16 [35] achieved the best effect in the precision, under-segmentation, IOU_B, and ASE.

However, its recall rate was also the lowest, resulting in no improvement in the IOU_M, the most comprehensive and important performance metric. On the other hand, both the U-Net+res18 (ours) and U-Net+res34 (ours) had good improvement over the U-Net+dd, however, the U-Net+res34 (ours) had no noticeable performance improvement over the U-Net+res18 (ours), even with worse performance in the ASE. Therefore, res18 was selected as our encoder for extracting features.

**Figure 8:**
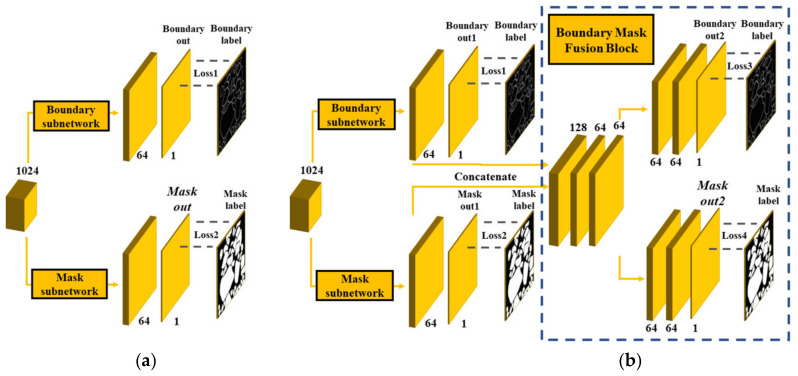
Difference of decoders with/without a boundary mask fusion block (BMFB); (**a**) the decoder of U-Net_DCAN [26]; and (**b**) the decoder of U-Net+dd.

In the design of the decoder, we conducted comparison experiments of different decoders (i.e., the original U-Net, deep contour-aware networks U-Net (U-Net_DCAN [26]) (without BMFB), and U-Net+dd). All encoders of the three models were the same as the original encoder of the U-Net. The difference of decoders between U-Net_DCAN [26] and U-Net+dd is shown in Figure 8. From the prediction results in Table 4, the U-Net_DCAN [26] had the worst prediction result on the testing dataset.

In contrast, the U-Net+dd had the best result, which indicates that information from the boundary subnetwork and mask subnetwork must be fused. The above conclusion can be drawn from the fact that even the performance of the original U-Net was better than that of U-Net_DCAN [26], showing that independently adding another decoder without BMFM is useless for mask prediction.

**Figure 9:**
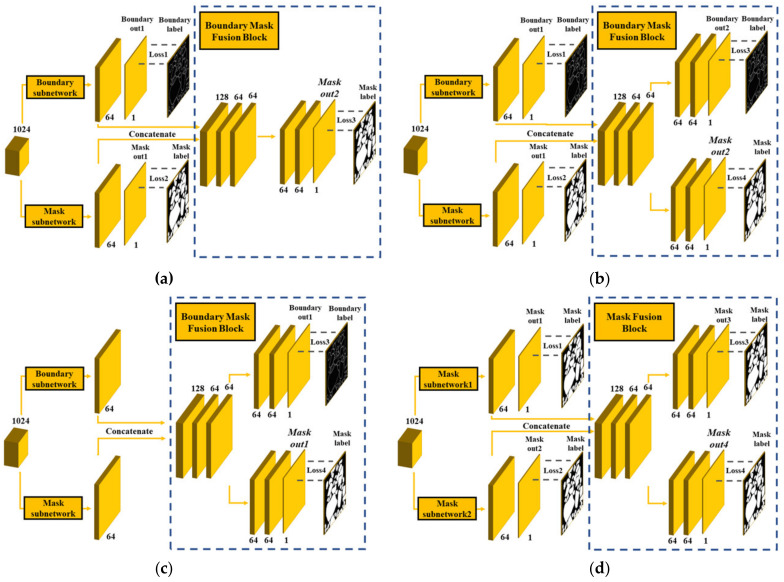
Simplified schematic diagram of the four models with different BMFBs: (**a**) the decoder of U-Net+res18+BA [27]; (**b**) the decoder of U-Net+res18 +dd; (**c**) the decoder of U-Net+res18+dd+loss3_4; and (**d**) the decoder of U-Net+res18+dd+mask.

We performed comparison experiments between the model with BMFB from [27], called Boundary-aware U-Net (U-Net+res18+BA [27]), in which only the mask subnetwork was used, and the model with our designed BMFB with boundary and mask subnetworks, named U-Net+res18+dd. In addition, the model U-Net+res18+dd+loss3_4, which is the same as the U-Net+res18+dd except that Loss1 and Loss2 are omitted, was compared with our model. The last model, U-Net+res18+dd+mask, is also the same as the U-Net+res18+dd except that all four losses are from mask labels. The simplified schematic diagram of the four models is shown in Figure 9. The “+res18” in this section also includes the pre-trained parameters from the ImageNet. The quantitative experiment results on the testing dataset are shown in Table 5.

The conclusion that can be drawn from Table 5 is that U-Net+res18+dd had better results than U-Net+res18+BA [27] in most performance metrics, proving the effectiveness of our designed BMFB with boundary and mask subnetworks. Table 5 also shows that the U-Net+res18+dd+loss3_4 had a relatively worse performance than U-Net+res18+dd, which demonstrates that Loss1 and Loss2 before BMFB are necessary for letting each subnetwork know what they need to learn.

By comparing the results of U-Net+res18+dd and U-Net+res18+dd+mask, the effectiveness of the boundary information was proved, showing that adding boundary information was more conducive to ore image segmentation. All results of the four models were better than those of the original U-Net, demonstrating the effectiveness of our designed res18 encoder and dd decoder.

Another interesting experiment was conducted to determine how to obtain the boundary labels. In [26,27], boundary labels were derived by dilating the canny edge results obtained from the ore mask labels. The boundary labels used in our previous experiments were canny edge labels. The U-Net+dd+canny means that boundary labels used were the canny edge ones, while the U-Net+dd+dilate indicates that the boundary labels were the ones obtained by dilating the canny edge labels in OpenCV.

**Figure 10:**
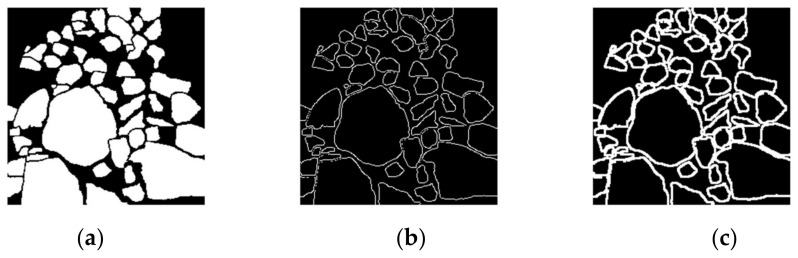
Mask label and two different boundary labels: (**a**) the mask label; (**b**) the canny boundary label; and (**c**) the dilated boundary label.

The annotation ore mask label, canny boundary label, and dilated boundary label are shown in Figure 10. Table 6 shows that the U-Net+dd_canny had better results in most performance metrics than the U-Net+dd_dilate. On the other hand, even the U-Net+dd_dilate had better results than the original U-Net, proving the effectiveness of our designed dd decoder in this paper.

Finally, our proposed model with the res18 encoder and dd decoder, which was initialized with the pre-trained parameters from ImageNet, was compared with the classic U-Net, fully convolutional networks (FCN8), and pyramid scene parsing network (PSP). The quantitative comparison results are shown in Table 7.

From Table 7, compared with U-Net, FCN8, and PSP, our proposed model achieved the best results in all performance metrics except for the inference time for one image using the single 1080Ti GPU. From the results of the U-Net, FCN8, and PSP, in particular with the IOU_B, with a unique symmetrical decoder that restored the resolution step by step, and a skip connection that connected the low-level position information and the high-level semantic information, the U-Net had an inherent advantage over FCN8 and PSP in ore segmentation, particularly regarding the boundary position.

With another decoding path for the boundary in our proposed model, whose information was sent to BMFB for fusion, the segmentation performance for ore (especially for the ore boundary) was further improved showing a 1.01% increase in IOU_M and a 5.78% increase in IOU_B over the original U-Net. As one of the best semantic segmentation methods at present, the performance of PSP in ore image segmentation was not better than that of FCN8, possibly because it paid more attention to global information due to its pyramid pooling module (PPM) and it ignored many small ores and ore boundary information, which affected the recall rate and led to a relatively low accuracy and IOU.

**Figure 11:**
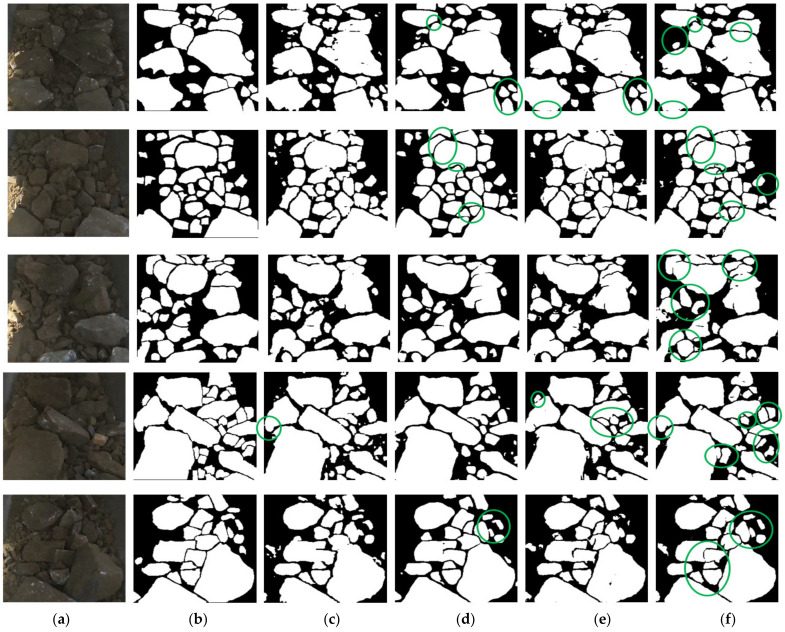
Prediction of different models: (**a**) the raw images; (**b**) the ground truth; (**c**) the results of FCN8; (**d**) the results of PSP; (**e**) the results of U-Net; and (**f**) the results of our model.

The prediction results of the four models are shown in Figure 11. FCN8 exhibited the worst performance. Compared with the U-Net, our proposed model was more conducive to segmenting adhesive ores, and has higher recall and precision. Thanks to the PPM, the PSP has a larger receptive field and can learn multi-scale information, which can alleviate the phenomenon of texture and shadow on the surface of large ores being predicted as the boundary and background. However, this comes with an inaccurate ore segmentation for the boundary and low recall rate for small ores in the image.

## 5. Conclusions

We proposed a novel multi-task semantic segmentation network based on U-Net for the conveyor belt ore image segmentation. To solve the problem of small training data and improve the feature extraction ability of the model, an improved encoder based on Resnet18, called res18, was presented. Compared with the original U-Net, the decoder of this model, named dd, included a boundary subnetwork for boundary detection and a mask subnetwork for mask segmentation, and the information from two subnetworks was fused in the BMFM.

The experimental results showed that the pixel accuracy, IOU_M, IOU_B, and ASE rate of our proposed model on the testing dataset were 92.07%, 86.95%, 52.32%, and 20.38%, respectively. Compared to U-Net, the improvements were 0.65%, 1.01%, 5.78%, and 12.11% (down), respectively. Specifically, our proposed model with re-designed dd was more conducive to segmenting adhesive ores, and this ability could be applied to medical tissue cell images and metal material microscopic images.

The drawback of our model is that it requires more parameters as it adds another decoder for boundary detection. To improve the performance of U-Net based models on ore image segmentation without adding additional parameters, possible work could be done regarding the loss function in the future, such as adding more weight to the ore boundary pixel loss. On the other hand, labeling ore images for semantic segmentation is highly time-consuming. To achieve the goal of detecting large ores and special-shaped ores, the object detection techniques, whose annotation work is relatively easy, can be applied at the mine site.

## Figures and Tables

**Table 1:** Network structure comparison between different encoders.

Layer Name	Output Size of the 3 Encoders [n×n]	Our Improved Encoder	Original ResNet18	Original U-Net Encoder
**Layer1_0**	256/64/---	3 × 3, 64, stride 1	7 × 7, 64, stride 2 3 × 3 max pool, stride 2	-
**Layer1**	256/64/256	$\left[\begin{array}{c} 3\times 3,64\\ 3\times 3,64 \end{array}\right]\times 2$	$\left[\begin{array}{c} 3\times 3,64\\ 3\times 3,64 \end{array}\right]\times 2$	3 × 3, 64 3 × 3, 64
**Layer2**	128/32/128	$\left[\begin{array}{c} 3\times 3,128\\ 3\times 3,128 \end{array}\right]\times 2$	$\left[\begin{array}{c} 3\times 3,128\\ 3\times 3,128 \end{array}\right]\times 2$	2 × 2 max pool, stride 2 3 × 3, 128 3 × 3, 128
**Layer3**	64/16/64	$\left[\begin{array}{c} 3\times 3,256\\ 3\times 3,256 \end{array}\right]\times 2$	$\left[\begin{array}{c} 3\times 3,256\\ 3\times 3,256 \end{array}\right]\times 2$	2 × 2 max pool, stride 2 3 × 3, 256 3 × 3, 256
**Layer4**	32/8/32	$\left[\begin{array}{c} 3\times 3,512\\ 3\times 3,512 \end{array}\right]\times 2$	$\left[\begin{array}{c} 3\times 3,512\\ 3\times 3,512 \end{array}\right]\times 2$	2 × 2 max pool, stride 2 3 × 3, 512 3 × 3, 512
**Layer5**	16/---/16	2 × 2 max pool, stride 2 3 × 3, 1024 3 × 3, 1024	fc layer	2 × 2 max pool, stride 2 3 × 3, 1024 3 × 3, 1024

**Table 2:** Comparison among different U-Net based models. Pixel accuracy (Acc), F1 score, Intersection over Union for the ore mask (IOU_M), precision, recall, under-segmentation, over-segmentation, Intersection over Union for the ore boundary (IOU_B), and error of the average statistical ore particle size (ASE). The bold number means the best result.

	Acc	F1	IOU_M	Precision	Recall	Under_seg	Over_seg	IOU_B	ASE
U-Net	91.41	92.42	85.94	92.91	92.04	9.00	7.96	46.54	32.49
U-Net+res18	91.46	92.48	86.04	92.91	92.16	9.19	7.80	47.54	30.33
U-Net+res18+param	91.81	92.88	86.74	91.94	**93.90**	10.84	**6.09**	49.44	30.43
U-Net+dd	91.67	92.63	86.33	93.55	91.83	**8.23**	8.16	48.86	22.13
U-Net+res18+dd	91.76	92.75	86.52	**93.59**	92.03	8.32	7.96	50.36	23.93
U-Net+res18+param+dd	**92.07**	**93.00**	**86.95**	93.46	92.64	8.50	7.36	**52.32**	**20.38**

**Table 3:** Comparison among different encoders. The bold number means the best result.

	Acc	F1	IOU_M	Precision	Recall	Under_seg	Over_seg	IOU_B	ASE
U-Net	91.41	92.42	85.94	92.91	92.04	9.00	7.96	46.54	32.49
U-Net+VGG16 [35]	91.41	92.20	85.56	**95.58**	89.14	**5.40**	10.86	48.78	**17.21**
U-Net+Res18 [30]	91.14	92.07	85.33	94.12	90.20	7.46	9.79	46.18	22.37
U-Net+dd	91.67	92.63	86.33	93.55	91.83	8.23	8.16	48.60	22.13
U-Net+res18 (ours)	**92.07**	**93.00**	**86.95**	93.46	**92.64**	8.50	**7.36**	52.32	20.38
U-Net+res34 (ours)	92.07	93.00	86.94	93.48	92.61	8.43	7.38	**52.72**	23.22

**Table 4:** Comparison of different models with/without a boundary mask fusion block (BMFB). The bold number means the best result.

	Acc	F1	IOU_M	Precision	Recall	Under_seg	Over_seg	IOU_B	ASE
U-Net	91.41	92.42	85.94	92.91	**92.04**	9.00	7.96	46.54	32.49
U-Net_DCAN [26]	91.04	92.14	85.46	92.28	92.11	10.16	**7.8**	46.30	37.17
U-Net+dd	**91.67**	**92.63**	**86.33**	**93.55**	91.83	**8.23**	8.16	**48.60**	**22.13**

**Table 5:** Model comparisons of different boundary mask fusion blocks (BMFBs). The bold number means the best result.

	Acc	F1	IOU_M	Precision	Recall	Under_seg	Over_seg	IOU_B	ASE
U-Net	91.41	92.42	85.94	92.91	92.04	9.00	7.96	46.54	32.49
U-Net+res18+BA [27]	91.57	92.68	86.41	91.89	**93.58**	10.91	**6.41**	51.60	33.35
U-Net+res18+dd	**92.07**	**93.00**	**86.95**	93.46	92.64	8.50	7.36	**52.72**	**20.38**
U-Net+res18+dd+loss3_4	91.61	92.58	86.23	93.05	92.21	9.08	7.70	50.35	32.29
U-Net+res18+dd+mask	91.68	92.52	86.13	**94.35**	90.84	**7.10**	9.10	48.98	26.01

**Table 6:** Comparison among different boundary labels. The bold number means the best result.

	Acc	F1	IOU_M	Precision	Recall	Under_seg	Over_seg	IOU_B	ASE
U-Net	91.41	92.42	85.94	92.91	**92.04**	9.00	**7.96**	46.54	32.49
U-Net+dd_canny	**91.67**	**92.63**	**86.33**	**93.55**	91.83	**8.23**	8.16	**48.60**	**22.13**
U-Net+dd_dilate	91.53	92.52	86.11	93.46	91.70	8.50	8.30	48.59	26.59

**Table 7:** Comparison among different models. The bold number means the best result.

	Acc	F1	IOU_M	Precision	Recall	Under_seg	Over_seg	IOU_B	ASE	Inference Time
U-Net	91.41	92.42	85.94	92.91	92.04	9.00	7.96	46.54	32.49	44.65ms
FCN8	90.73	91.84	84.94	91.88	91.91	10.60	8.08	37.36	46.33	**22.81ms**
PSP	90.48	91.61	84.54	92.39	90.90	9.97	9.09	34.18	28.16	38.33ms
Ours	**92.07**	**93.00**	**86.95**	**93.46**	**92.64**	**8.50**	**7.36**	**52.32**	**20.38**	105.31ms

## Data Availability

Not applicable.
